# *Phaeodactylum tricornutum* photorespiration takes part in glycerol metabolism and is important for nitrogen-limited response

**DOI:** 10.1186/s13068-015-0256-5

**Published:** 2015-05-03

**Authors:** Aiyou Huang, Lixia Liu, Chen Yang, Guangce Wang

**Affiliations:** Institute of Oceanology, Chinese Academy of Sciences, Qingdao, 266071 China; Institute of Plant Physiology and Ecology, Shanghai Institute for Biological Sciences, Chinese Academy of Sciences, Shanghai, 200032 China

**Keywords:** *Phaeodactylum tricornutum*, Photorespiration, Glycerol metabolism, Nitrogen-limited response

## Abstract

**Background:**

Microalgae are potential sources of biofuels and high-value compounds. Mixotrophic conditions usually promote growth of microalgae. The pennate diatom *Phaeodactylum tricornutum*, with its short life cycle, completely sequenced genome, and ease of transformation, can be used as a model for studying carbon metabolism in microalgae.

**Results:**

We compared the growth rate of *P. tricornutum* (IOCAS-001) under different conditions and labeled the cells using [^13^C]glycerol (GL). The results revealed GL promoted the growth of *P. tricornutum*. Ser and Gly were synthesized via photorespiration. The ^13^C enrichment of Ser and Gly under nitrogen-limited conditions was much higher compared to other amino acids, indicating the enhancement of photorespiration. Addition of sodium acetate decreased the growth rate of *P. tricornutum* under nitrogen-limited conditions. Our results indicated that the GL carbon backbone enters the Calvin cycle in the form of dihydroxyacetone phosphate (DHAP), producing xylulose 5-phosphate (X5P) with a GL2_3-generated carbon backbone distributed at X5P1_2 and ribose 5-phosphate (R5P) with GL1-derived carbon atoms at R5P1 and R5P2. Both R5P and X5P can be converted into ribulose-1,5-bisphosphate (RuBP). By oxygenation of RuBP carboxylase/oxygenase (Rubisco) and metabolism through photorespiration, these RuBPs generate Ser and Gly with GL1 or GL2-derived carbon atoms at position 1 and GL1 or GL3-derived carbon atoms at other positions, resulting in a low level of ^13^C enrichment of Gly1 and Ser1.

**Conclusion:**

Our results indicated different strains of *P. tricornutum* have different mechanisms for organic carbon metabolism. Photorespiration is involved in GL metabolism and is important for the nitrogen-limited response in *P. tricornutum*.

**Classification:**

Metabolic flux analysis, microalgae

**Electronic supplementary material:**

The online version of this article (doi:10.1186/s13068-015-0256-5) contains supplementary material, which is available to authorized users.

## Introduction

Microalgae have received increasing attention as potential sources of biofuels, high-value chemicals, pharmaceuticals, bioactive compounds, and so on. Variations of culture conditions, including carbon source, have been investigated in attempts to promote the production of high-value compounds. Microalgae cultivated under mixotrophic conditions usually have higher growth rate and accumulate greater biomass as well as specific high-value compounds at the plateau phase compared to photoautotrophic conditions [1-3]. Acetate, for example, enhanced carotenoid biosynthesis in the late-exponential growth phase in *Haematococcus pluvialis* [4], and glucose, the common carbohydrate in bacterial culture, can be used by *Chlorella* and promotes the growth rate significantly [5]. A clear illustration of carbon metabolism, especially utilization of organic substrates and the mechanism underlying accumulation of high-value compounds, is important for industrialization of microalgae production. Related studies should be conducted, especially in model organisms.

Diatoms, which are believed to produce around one-fifth of the primary productivity on earth [6,7], can produce approximately 46 tons of organic oil ha^−1^·year^−1^ and are, therefore, potential sources of biodiesel fuel [8]. The pennate diatom *Phaeodactylum tricornutum* is an atypical diatom with a weakly silicified outer shell [9-12] that is ruptured easily by sonication or high-pressure homogenization, facilitating extraction of intracellular metabolites. *P. tricornutum* is a potential source of polyunsaturated fatty acids (PUFA) [13] and fucoxanthin [14], the marine carotenoid reported to exert anti-carcinogenic effects [15] as well as radical scavenging [16] and, most excitingly, to have anti-obesity properties [17,18]. The *P. tricornutum* (CCMP 2561) genome has been sequenced completely, providing a clear genetic background and revealing *P. tricornutum* adopts the metabolic pathways of both plants and animals [19]. *P. tricornutum* is easily transformed and can be regulated genetically [20-23]. Because of the characteristics mentioned above, *P. tricornutum* is considered to be a potential source for biodiesel and the production of high-value compounds, as well as a model for studying carbon metabolism in microalgae.

*P. tricornutum* is a photoautotrophic organism capable of photosynthesis using light energy and assimilating inorganic carbon sources, like plant cells. Also, there are reports of *P. tricornutum* using organic substrates as carbon and energy sources under mixotrophic conditions. The idea that *P. tricornutum* can use glycerol is quite widely known [1-3,24], whereas reports of its ability to use glucose were controversial [25,26]. To demonstrate a clear pathway for organic carbon metabolism in *P. tricornutum*, methods such as metabolic flux analysis can be used. Stationary metabolic flux analysis used ^13^C-labeled carbon source as substrate. During metabolism, the carbon backbone of the substrate is broken and recombined in different pathways, producing metabolites with different labeling patterns that can be detected through gas chromatography mass spectrometry (GC-MS) or nuclear magnetic resonance (NMR). These data can be used to calculate the contributions of different pathways to the synthesis of a defined metabolite, that is, flux ratios. Combined with a stoichiometric reaction model and extracellular consumption and secretion rates, these flux ratios can be used to quantify the net carbon fluxes in central carbon metabolism [27]. Based on ^13^C labeling experiment using glucose and glycerol as carbon sources, Zheng *et al.* found unusual pathways were active in *P. tricornutum* (CCMP 632). Ser and Gly were largely synthesized via the glyoxylate (GOC) cycle followed by photorespiratory reactions [26]. This provided important information about glucose metabolism in *P. tricornutum* (CCMP 632). Nevertheless, some previous studies had reported that *P. tricornutum* cannot utilize glucose at all [25], indicting that different strains of *P. tricornutum* have different efficiencies of glucose metabolism. Strains that lack a glucose-utilizing ability might have a different mechanism for organic carbon metabolism from strains that can use glucose.

In this study, we compared the growth rates of *P. tricornutum* (IOCAS-001) under different conditions and labeled cells with [^13^C]glycerol (GL). The results revealed the strain in this study cannot use glucose. Ser and Gly were synthesized from GOC that origin from photorespiration but not the GOC cycle. Photorespiration was indeed involved in GL metabolism and was important for the nitrogen-limited response in *P. tricornutum*.

## Results

### Growth of *P. tricornutum* under different culture conditions

#### Influence of carbon source

**Figure 1:**
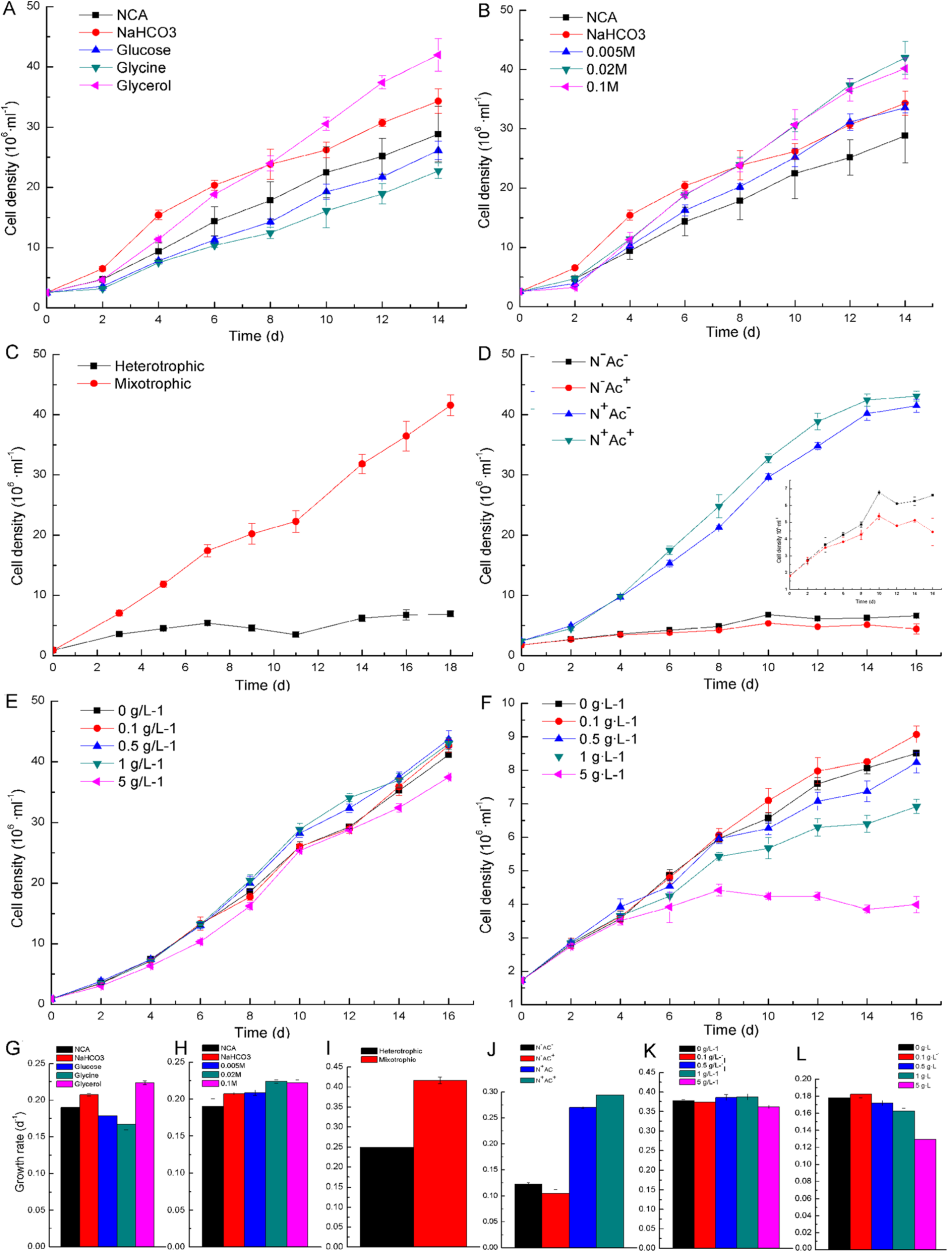
Growth of *P. tricornutum* on different conditions. Cell densities and growth rates of *P. tricornutum* grown on different carbon sources **(A**, **G)**, under different GL concentrations **(B**, **H)**, under mixotrophic and heterotrophic conditions **(C, I)**, in the presence or absence of sodium acetate **(D**, **J)**, under different sodium acetate concentrations in the presence of nitrogen **(E**, **K)**, and under different sodium acetate concentrations in the absence of nitrogen **(F**, **L)**. A-F *Y* axis, cell density (10^6^·mL^−1^), *X* axis, culture time; G-L *Y* axis, growth rate (day^−1^); NAC, no additional carbon source; N^+^Ac^+^, both sodium nitrate and sodium acetate plus; N^+^Ac^−^, sodium nitrate alone; N^−^Ac^+^, sodium acetate alone; N^−^Ac^−^, both sodium nitrate and sodium acetate minus. Error bars are the SD of three replicates.

We compared the growth rate of *P. tricornutum* (IOCAS-001) under conditions of no additional carbon source, 0.174 g·L^−1^ NaHCO_3_, 0.02 M glucose, 0.02 M GL, and 0.02 M glycine. The growth rate of *P. tricornutum* on 0.02 M glucose or 0.02 M glycine was significantly lower (*P* <0.01) compared to 0.174 g·L^−1^ NaHCO_3_ and even lower compared to that with no additional carbon source (Figure 1A,G). The growth rate of *P. tricornutum* with 0.02 M GL was higher (*P* <0.05) compared to no additional carbon source and even higher (*P* <0.05) than 0.174 g·L^−1^ NaHCO_3_ after 10 days of culturing (Figure 1A,G), indicating addition of GL promoted the growth of *P. tricornutum*, whereas glucose or glycine might not be utilized as carbon sources by *P. tricornutum*.

#### Influence of GL concentration

Because ^13^C-labeling GL is expensive, we optimized the concentration of GL to save substrate without affecting the growth rate of *P. tricornutum*. We compared the growth rate of *P. tricornutum* under conditions of no additional carbon source, 0.174 g·L^−1^ NaHCO_3_, 0.005 M, 0.02 M, and 0.1 M GL. The results showed the growth rate of *P. tricornutum* on 0.005 M GL and with 0.174 g·L^−1^ NaHCO_3_ were similar, whereas the growth rate of *P. tricornutum* on 0.02 M and 0.1 M GL were similar and higher (*P* <0.01) compared to 0.174 g·L^−1^ NaHCO_3_ (Figure 1B,H), indicating 0.02 M GL was sufficient for the growth of *P. tricornutum*. We chose 0.02 M GL as the substrate for mixotrophic growth of *P. tricornutum*.

#### Influence of light

Using 0.02 M GL as the carbon source, we compared the growth rate of *P. tricornutum* under mixotrophic conditions (that is, GL assimilated simultaneously with CO_2_ fixation under cool white fluorescent lights with a 12 h dark/12 h light cycle) and heterotrophic conditions (no light during culture). The results showed *P. tricornutum* can used glycerol to sustain growth under heterotrophic conditions, though the growth rate was much lower (*P* <0.01) compared to under mixotrophic conditions (Figure 1C,I), indicating light was important for growth of *P. tricornutum* even in the presence of GL as carbon source.

#### Influence of sodium acetate

To investigate the likely influence of sodium acetate on the growth rate of *P. tricornutum*, we compared the growth of *P. tricornutum* under different conditions (both sodium nitrate and sodium acetate plus, N^+^Ac^+^; sodium nitrate alone, N^+^Ac^−^; sodium acetate alone, N^−^Ac^+^; both sodium nitrate and sodium acetate minus, N^−^Ac^−^). The results indicted that in the presence of sodium nitrate, addition of sodium acetate can promote (*P* <0.01) the growth of P. tricornutum (Figure 1D,J). While under sodium nitrate minus condition, addition of sodium acetate limited (*P* <0.01) the growth of P. tricornutum (Figure 1D,J).

To further investigate the dose-response effect of acetate on growth rate of *P. tricornutum*, sodium acetate was added to different concentrations (0, 0.1, 0.5, 1, and 5 g·L^−1^) under sodium nitrate plus or minus conditions. The results indicted that, compared to acetate minus conditions, 0.1 g·L^−1^ sodium acetate had a negligible influence on growth of *P. tricornutum* under both sodium nitrate plus and minus conditions, 0.5 and 1 g·L^−1^ sodium acetate had a positive influence (*P* <0.05) under nitrate plus conditions and a negative effect (*P* <0.05) under nitrate minus conditions, while 5 g·L^−1^ sodium acetate limited (*P* <0.01) the growth of *P. tricornutum* under both nitrate plus and minus conditions (Figure 1E,F,K,L).

### Change in glycerol and acetate concentration

**Figure 2:**
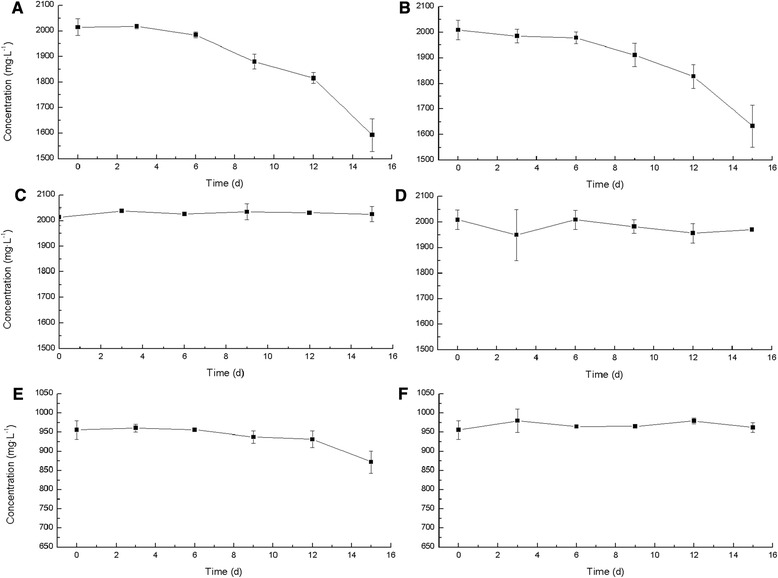
Change in glycerol and acetate concentrations under different conditions. Change of glycerol concentration under N^+^Ac^+^ **(A)**, N^+^Ac^−^ **(B)**, N^−^Ac^+^ **(C)**, and N^−^Ac^−^ **(D)** conditions and change of sodium acetate concentration under N^+^Ac^+^ **(E)** and N^−^Ac^+^ **(F)** conditions.

To verify the consumption of glycerol and acetate, the supernatants from cultures under N^+^Ac^+^, N^+^Ac^−^, N^−^Ac^+^, and N^−^Ac^−^ conditions were collected, and glycerol and acetate concentrations were determined. There was a significant decrease in glycerol concentration under N^+^Ac^+^ and N^+^Ac^−^ conditions (from approximately 2000 to approximately 1600 mg · L^−1^, Figure 2A,B), while the consumption of glycerol under N^−^Ac^+^ and N^−^Ac^−^ conditions was not significant (Figure 2C,D). Similarly, the consumption of acetate was significant under N^+^Ac^+^ conditions (from approximately 960 to approximately 870 mg · L^−1^ sodium acetate, Figure 2E) but not obvious under N^−^Ac^+^ conditions (Figure 2F). It was likely that the decrease in glycerol and acetate concentrations under N^-^ condition was too slight to be detected. As the biomass did not increase much under N^-^ condition, possibly only a little glycerol and acetate were consumed.

### Total lipid content

**Figure 3:**
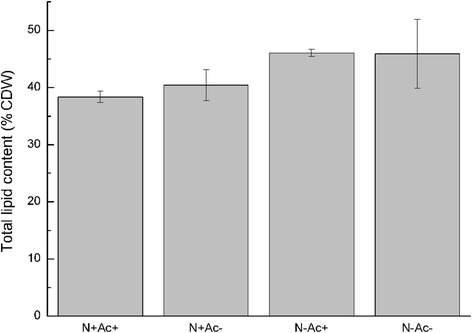
Total lipid contents under N^+^Ac^+^, N^+^Ac^−^, N^−^Ac^+^, and N^−^Ac^−^ conditions.

Total lipid content was determined to verify if consumption of acetate influenced synthesis of total lipid. Though acetate was consumed under N^+^ Ac^+^ conditions, there was no significant difference between total lipid contents of N^+^Ac^+^ and N^+^Ac^−^ (Figure 3). There was no significant difference between total lipid contents of N^−^Ac^+^ and N^−^Ac^−^, either (Figure 3). Total lipid contents range from approximately 38% to approximately 46% cell dry weight (CDW).

### ^13^C-labeling experiment

#### *Acclimation of* P. tricornutum *to an organic carbon source under mixotrophic conditions*

To investigate the metabolism of GL in *P. tricornutum*, we cultured *P. tricornutum* using [^13^C]GL and detected the distribution of ^13^C enrichments in amino acids. At first, the cultures used as inocula were grown on f/2 medium with NaHCO_3_ as carbon source, and the level of [U-^13^C]GL was 20%. Table 1 gives the corrected abundance of the mass isotopomers for hydrolyzed amino acids in 20% [U-^13^C]GL labeling experiments; the mean fractional labeling (FL) was approximately 0.047. As the ^13^C isotope is stable and the natural abundance is 0.011 [28], the experimental error of some fragments, which were slightly higher than the natural ^13^C abundance, might be large. Thus, these data were not analyzed further. We suggested the reason might be because the cultures used as inocula were cultivated with NaHCO_3_ as carbon source, and the cell might need time for acclimation before it can utilize GL when transferred to medium with GL as carbon source.

**Table 1:** **FLs for amino acids under different labeling experiments**

**Fragments**	**20% U-** ^**13**^ **C** ^**a**^	**30% U-** ^**13**^ **C N +** ^**b**^	**30% U-** ^**13**^ **C N-** ^**c**^	**1,3-** ^**13**^ **C N +** ^**d**^	**1,3-** ^**13**^ **C N-** ^**e**^
M_ala_057	0.057 ± 0.003	0.180 ± 0.015	0.122 ± 0.006	0.190 ± 0.012	0.161 ± 0.005
M_ala_085	0.076 ± 0.001	0.195 ± 0.013	0.143 ± 0.005	0.202 ± 0.009	0.180 ± 0.004
M_asx_057	0.041 ± 0.001	0.146 ± 0.011	0.094 ± 0.003	0.151 ± 0.016	0.119 ± 0.003
M_asx_085	0.045 ± 0.001	0.149 ± 0.012	0.097 ± 0.003	0.159 ± 0.016	0.125 ± 0.004
M_asx_302	0.042 ± 0.001	0.149 ± 0.010	0.097 ± 0.003	0.148 ± 0.016	0.115 ± 0.004
M_glx_057	0.046 ± 0.001	0.168 ± 0.015	0.140 ± 0.001	0.175 ± 0.013	0.155 ± 0.005
M_glx_085	0.047 ± 0.002	0.170 ± 0.017	0.144 ± 0.004	0.183 ± 0.013	0.165 ± 0.003
M_glx_302	0.068 ± 0.004	0.170 ± 0.014	0.154 ± 0.022	0.157 ± 0.007	0.157 ± 0.015
M_gly_057	0.049 ± 0.002	0.172 ± 0.013	0.294 ± 0.025	0.190 ± 0.008	0.219 ± 0.002
M_gly_085	0.050 ± 0.003	0.173 ± 0.014	0.292 ± 0.025	0.254 ± 0.006	0.362 ± 0.004
M_his_057	-	0.127 ± 0.006	-	0.107 ± 0.029	0.103 ± 0.036
M_his_159	-	0.144 ± 0.026	-	0.127 ± 0.013	0.109 ± 0.008
M_his_302	-	0.134 ± 0.010	-	0.121 ± 0.015	0.096 ± 0.021
M_ile_015	0.041 ± 0.001	0.128 ± 0.006	0.047 ± 0.026	0.124 ± 0.012	0.072 ± 0.043
M_ile_085	0.048 ± 0.002	0.133 ± 0.005	0.069 ± 0.001	0.137 ± 0.014	0.109 ± 0.001
M_leu_015	0.041 ± 0.001	0.135 ± 0.005	0.060 ± 0.013	0.136 ± 0.013	0.109 ± 0.004
M_leu_085	0.047 ± 0.002	0.141 ± 0.005	0.069 ± 0.003	0.150 ± 0.014	0.120 ± 0.002
M_lys_057	0.042 ± 0.002	0.117 ± 0.006	0.073 ± 0.024	0.117 ± 0.012	0.090 ± 0.000
M_lys_159	0.051 ± 0.004	0.119 ± 0.006	0.089 ± 0.046	0.121 ± 0.016	0.095 ± 0.001
M_lys_302	0.064 ± 0.010	0.129 ± 0.005	0.078 ± 0.072	0.092 ± 0.009	0.087 ± 0.002
M_met_057	0.000 ± 0.000	0.148 ± 0.011	0.106 ± 0.031	0.163 ± 0.014	0.161 ± 0.003
M_met_085	0.000 ± 0.000	0.150 ± 0.011	0.142 ± 0.009	0.180 ± 0.017	0.179 ± 0.010
M_phe_057	0.050 ± 0.001	0.142 ± 0.006	0.069 ± 0.006	0.140 ± 0.013	0.118 ± 0.026
M_phe_085	0.048 ± 0.002	0.143 ± 0.006	0.073 ± 0.015	0.139 ± 0.014	0.101 ± 0.013
M_phe_302	0.045 ± 0.003	0.137 ± 0.009	0.056 ± 0.003	0.111 ± 0.012	0.073 ± 0.004
M_phe_sc	0.098 ± 0.002	0.196 ± 0.008	0.366 ± 0.165	0.198 ± 0.014	0.339 ± 0.191
M_pro_057	0.044 ± 0.001	0.140 ± 0.011	0.119 ± 0.018	0.158 ± 0.017	0.121 ± 0.008
M_pro_085	0.042 ± 0.001	0.148 ± 0.012	0.106 ± 0.005	0.162 ± 0.020	0.119 ± 0.001
M_ser_057	0.057 ± 0.001	0.183 ± 0.012	0.313 ± 0.011	0.225 ± 0.009	0.257 ± 0.001
M_ser_085	0.058 ± 0.001	0.184 ± 0.012	0.303 ± 0.014	0.269 ± 0.008	0.346 ± 0.005
M_ser_302	0.057 ± 0.001	0.185 ± 0.012	0.323 ± 0.018	0.199 ± 0.008	0.211 ± 0.001
M_thr_057	0.041 ± 0.001	0.124 ± 0.008	0.070 ± 0.002	0.128 ± 0.017	0.095 ± 0.001
M_thr_085	0.040 ± 0.005	0.125 ± 0.008	0.077 ± 0.006	0.132 ± 0.014	0.100 ± 0.001
M_thr_sc	0.117 ± 0.003	0.180 ± 0.008	0.155 ± 0.030	0.188 ± 0.014	0.167 ± 0.014
M_val_057	0.047 ± 0.002	0.145 ± 0.006	0.073 ± 0.004	0.150 ± 0.013	0.109 ± 0.003
M_val_085	0.054 ± 0.002	0.152 ± 0.005	0.087 ± 0.007	0.152 ± 0.013	0.118 ± 0.002
M_val_302	0.049 ± 0.001	0.152 ± 0.005	0.080 ± 0.005	0.138 ± 0.013	0.101 ± 0.004
M_tyr_057	0.048 ± 0.002	0.140 ± 0.005	0.063 ± 0.005	0.140 ± 0.013	0.103 ± 0.019
M_tyr_085	0.053 ± 0.001	0.143 ± 0.005	0.096 ± 0.041	0.140 ± 0.013	0.150 ± 0.098
M_tyr_302	0.047 ± 0.003	0.140 ± 0.010	0.064 ± 0.005	0.112 ± 0.011	0.078 ± 0.006
average FL	0.047 ± 0.005	0.150 ± 0.008	0.121 ± 0.005	0.157 ± 0.014	0.141 ± 0.007

^a^FLs for amino acids under 20% [U-^13^C]glycerol labeling experiments, cultures with NaHCO_3_ as carbon source was used as inocula; ^b^FLs for amino acids under 30% [U-^13^C]glycerol labeling experiments, nitrogen plus; ^c^FLs for amino acids under 30% [U-^13^C]glycerol labeling experiments, nitrogen minus; ^d^FLs for amino acids under 50% [1,3-^13^C]glycerol labeling experiments, nitrogen plus; ^e^FLs for amino acids under 50% [1,3-^13^C]glycerol labeling experiments, nitrogen minus.

Next, cultures used for inocula were acclimated to organic carbon source for approximately 3 months by repeated transfer of cells in the mid-exponential growth phase into fresh medium with GL as carbon source, and the level of [U-^13^C]GL was increased to 30%. Table 1 gives the FL for hydrolyzed amino acids in 30% [U-^13^C]GL labeling experiments; the mean FL was approximately 0.150. Fragments from Phe and Tyr, which are generally synthesized from phosphoenolpyruvate (PEP) and erythrose-4-P (E4P), had similar values of mass distribution vector (MDV) (M_Phe_057, M_Phe_085, and M_Phe_302 *versus* M_Tyr_057, M_Tyr_085, and M_Tyr_302), confirming the consistency of GC-MS results.

#### The FL values of amino acids synthesized in different cell partitions were diluted to different degrees

Under mixotrophic conditions, different amino acid fragments had different FL values (Table 1) regardless of the type of labeling substrates ([U-^13^C]GL or [1,3-^13^C]GL). Eight amino acids (Lys, Thr, Ile, Leu, Tyr, His, Phe, and Val), which were reported to be synthesized in chloroplasts in higher plants, mainly had mostly lower average FL values, whereas average *FL* values for Ala, Ser, Gly, and Glx were higher (Table 2). This indicated GL was assimilated simultaneously with CO_2_ fixation in chloroplasts, resulting in different dilutions of FL values between amino acids synthesized in different cell partitions.

**Table 2:** **Average FL values for hydrolyzed amino acids in nitrogen plus (U-** ^**13**^ **C) labeling experiment**

**AA**	**FL ± SD**
Lys	0.118 ± 0.006
Thr	0.124 ± 0.008
Ile	0.130 ± 0.005
Leu	0.138 ± 0.005
Tyr	0.141 ± 0.006
His	0.135 ± 0.015
Phe	0.140 ± 0.007
Val	0.149 ± 0.005
Asx	0.148 ± 0.011
Met	0.149 ± 0.011
Pro	0.148 ± 0.012
Gly	0.172 ± 0.014
Glx	0.169 ± 0.015
Ser	0.184 ± 0.012
Ala	0.188 ± 0.014

FL, fractional labeling; SD, standard deviation.

#### ^13^C enrichment of Ser and Gly in the absence of nitrogen

As shown in Table 1, under nitrogen plus conditions, Ala had the highest average FL value followed by Ser, Gly, and Glx. In the absence of nitrogen, FL values of Ser and Gly were increased significantly with levels close to the ^13^C enrichment of substrate, indicating the Ser and Gly metabolic pathway was important for the nitrogen-limitation response in *P. tricornutum*.

#### Positional labeling of Ser and Gly based on [U-^13^C]GL or [1,3-^13^C]GL

We calculated the positional labeling of Ser and Gly to investigate the likely pathway for Ser and Gly metabolism in *P. tricornutum*. When [U-^13^C]GL was used as substrate, both Ser and Gly had similar ^13^C enrichment in all positions, regardless of the nitrogen conditions (Table 3). When [1,3-^13^C]GL was used as substrate in the presence of nitrogen, the ^13^C enrichment of Ser1 was significantly lower (approximately half) compared to Ser2 and Ser3, and the ^13^C enrichment of Gly1 was also approximately half compared to Gly2 (Table 3). Moreover, the ^13^C enrichment of Ser1 and Gly1 was decreased significantly in the absence of nitrogen, suggesting the majority of Ser1 and Gly1 were derived from GL2, consistent with the report of Zheng *et al.* [26].

**Table 3:** **Positional labeling of Ser and Gly**

	**U-** ^**13**^ **C, N plus**	**U-** ^**13**^ **C, N minus**	**1,3-** ^**13**^ **C, N plus**	**1,3-13C, N minus**
Ser1	0.175 ± 0.016	0.332 ± 0.006	0.137 ± 0.012	0.080 ± 0.010
Ser2	0.182 ± 0.010	0.313 ± 0.033	0.261 ± 0.005	0.341 ± 0.010
Ser3	0.175 ± 0.017	0.293 ± 0.010	0.276 ± 0.011	0.351 ± 0.001
Gly1	0.167 ± 0.011	0.295 ± 0.025	0.126 ± 0.010	0.076 ± 0.003
Gly2	0.169 ± 0.014	0.292 ± 0.025	0.254 ± 0.006	0.362 ± 0.004
Average FLs of AA	0.150 ± 0.008	0.121 ± 0.005	0.157 ± 0.014	0.141 ± 0.007
Substance FLs	0.3	0.3	0.33	0.33

FL, fractional labeling.

### Change in intracellular metabolite pools in response to nitrate limitation and acetate addition

Table 4 shows the ratios of abundances of key cellular metabolites under different culture conditions (N^−^Ac^+^, N^−^Ac^−^, N^+^Ac^+^, and N^+^Ac^−^). Gly, ethylene glycol, and formate increased under N^−^ conditions (N^−^Ac^+^/N^+^Ac^+^ and N^−^Ac^−^/N^+^Ac^−^) and were reduced when acetate was added (N^−^Ac^+^/N^−^Ac^−^ and N^+^Ac^+^/N^+^Ac^−^). Glu and Gln were reduced under N^−^ conditions (N^−^Ac^+^/N^+^Ac^+^ and N^−^Ac^−^/N^+^Ac^−^) and increased when acetate was added (N^−^Ac^+^/N^−^Ac^−^ and N^+^Ac^+^/N^+^Ac^−^). Accordingly, alpha ketoglutarate acid (AKG), the product of deamidization of Glu, increased under N^−^ conditions (N^−^Ac^+^/N^+^Ac^+^ and N^−^Ac^−^/N^+^Ac^−^) and was reduced when acetate was added (N^−^Ac^+^/N^−^Ac^−^ and N^+^Ac^+^/N^+^Ac^−^). This was in accord with the previous reports [29]. All amino acids, except Gly and *N*-Isovaleroylglycine, were reduced under N^−^ conditions (N^−^Ac^+^/N^+^Ac^+^ and N^−^Ac^−^/N^+^Ac^−^). Besides, intermediate products of orrnithine-urea cycle, including Asp, fumarate, and orrnithine, were reduced under N^−^ conditions (N^−^Ac^+^/N^+^Ac^+^ and N^−^Ac^−^/N^+^Ac^−^), in accord with the previous reports [30].

**Table 4:** **Ratios of abundances of key cellular metabolites under different culture conditions determined by NMR**

**Group**	**Metabolite**	**N-AC+ / N-AC-**	**N-AC+ / N + AC+**	**N + AC+ / N + AC-**	**N-AC- / N + AC-**
Alcohols	Ethanol	1.049*	1.067*	1.063	1.081
	Ethylene glycol	0.878	2.005*	1.293*	2.952**
Amines	Dimethylamine	1.153*	1.032	1.252*	1.120*
	Methylamine	1.143*	1.048	0.730**	0.670**
	Trimethylamine	0.804*	0.573**	1.140	0.812*
Amino acids	Alanine	1.176**	0.045**	0.965	0.037**
	Asparagine	-	0.000**	0.569**	0.000**
	Aspartate	-	0.000**	1.236**	0.000**
	Betaine	0.485**	0.385**	0.768**	0.608**
	Carnitine	1.079*	0.931	0.997	0.860*
	Glutamate	1.147*	0.257**	1.069	0.240**
	Glutamine	-	0.000**	1.220**	0.000**
	Glycine	0.657	1.241*	0.956	1.805*
	Isoleucine	0.964	0.168**	1.108	0.193**
	Leucine	0.876**	0.278**	0.880	0.279**
	Methionine	1.278*	0.067**	1.067	0.056**
	*N*-Isovaleroylglycine	1.333**	2.952**	1.448*	3.207**
	*O*-Acetylcarnitine	0.871**	0.853	1.107	1.083*
	Ornithine	-	0.000**	0.993	0.000**
	Phenylalanine	0.882*	0.462**	1.073	0.561**
	Proline	-	0.000**	1.177	0.000**
	Threonine	-	0.000**	1.516**	0.000**
	Tyrosine	0.959	0.678**	1.040	0.735**
	Valine	1.283**	0.154**	1.001	0.120**
Amino acid derivatives	Creatine	1.296**	1.658**	1.192**	1.526**
	Guanidoacetate	1.198*	0.330**	0.833	0.229**
Ammoniums Compounds	Choline	0.849*	0.028**	2.379**	0.080**
	*O*-Phosphocholine	0.790**	1.985**	0.712**	1.789**
	sn-Glycero-3-phosphocholine	0.945	1.180	0.975	1.218
Ketones	Acetone	0.578**	1.381**	0.647	1.547*
Methanol	Methanol	0.944	1.052	1.023	1.142*
Nucleic acid components	ATP	0.000**	0.000**	0.797	0.335**
	NAD+	1.002	-**	0.000	0.960
Organic acids	2-Oxoglutarate	0.874	22.509**	0.849	21.861**
	Acetate	1.077*	1.355*	1.079	1.357**
	Ascorbate	0.587**	0.327**	1.465*	0.817
	Formate	0.654**	1.099*	0.901	1.515**
	Fumarate	0.778**	0.462**	1.161	0.690*
	Isocitrate	-	-	0.000**	0.000**
	Lactate	0.586**	6.185**	2.303**	24.313**
	Pyruvate	0.938	0.455**	1.414	0.685**
	Succinate	0.694**	0.514**	0.991	0.734**
Sugars	Glucose	1.203	1.303*	1.341**	1.453**
	Glucose-1-phosphate	0.916	-**	-	-**
	Glucose-6-phosphate	1.210*	2.031**	1.200**	2.014**
	Glycerol	0.823**	0.595**	1.022	0.739**
Others	Trigonelline	1.098*	0.859**	1.122	0.878

**Student *t* test *P* value <0.01, *Student *t* test *P* value <0.05, ^−^Divide by zero.

## Discussion

### *P. tricornutum* use of organic carbon

In this study, the growth rates of *P. tricornutum* with 0.02 M glucose, 0.02 M GL, and 0.02 M glycine were compared to those with 0.174 g·L^−1^ NaHCO_3_ and no additional carbon source. The growth rate of *P. tricornutum* on 0.02 M glucose or 0.02 M glycine was significantly lower (*P* <0.01) compared to 0.174 g·L^−1^ NaHCO_3_ and even lower compared to no additional carbon source (Figure 1G), but the growth rate of *P. tricornutum* on 0.02 M GL was higher (*P* <0.01) compared to 0.174 g·L^−1^ NaHCO_3_ after 10 days of culture (Figure 1A). These results indicated GL can promote the growth of *P. tricornutum* significantly, whereas glucose and glycine might not be utilized by the *P. tricornutum* strain used in this study. The complete genome sequence revealed the *P. tricornutum* (CCMP 2561) genome lacks glucose permease (protein-Npi-phospho-l-histidine-sugar Npi-phosphotransferase, EC:2.7.1.69) [19], a class of enzymes that catalyze the phosphorylation of glucose and is important for utilization of extracellular glucose by cells. It is likely that the absence of glucose permease results in the poor glucose utilization ability of *P. tricornutum* (IOCAS-001). For the slow growth rate on glycine, we proposed the ammonium produced during the metabolism of glycine might be toxic to *P. tricornutum* cells because *P. tricornutum* growing on NH_4_Cl also has a slow growth rate (Additional file 1: Figure S1) in this study. Yet there are reports, including the report of Zheng *et al.*, that the mixotrophic growth rate of *P. tricornutum* on glucose or glycine is significantly higher compared to photoautotrophic conditions and accumulated a greater biomass at the plateau phase [1-3,26]. The inconsistent results about glucose utilization of *P. tricornutum* might be due to the use of different trains and different culture conditions. The strain fully sequenced was the *P. tricornutum* accession Pt1 8.6 deposited as CCMP2561 in the Provasoli-Guillard National Center for Culture of Marine Phytoplankton. The strain used by Zheng *et al.* was the *P. tricornutum* strain CCMP 632 obtained from the Provasoli-Guillard National Center for Marine Algae and Microbiota (NCMA) (East Boothbay, ME). The strain used in our study was screened from the East China Sea and maintained in our laboratory for decades and identified as *P. tricornutum* (IOCAS-001) according to 18s ribosomal RNA sequence. Thus, these strains are of different origins. It is probable that these strains are different in genome structure or have mutated (the uptake of a mobile genetic element that carries the genes necessary for glucose utilization or strain mutation over many sub-cultures) and resulted in different glucose utilization abilities. Further, experimental conditions might affect the results. Associated bacteria, for example, might help in the phosphorylation of glucose and thus help in the use of glucose by *P. tricornutum*. The *P. tricornutum* cultures used in this study were purified by growth on agar plates containing multi-antibiotics and shown to be bacteria-free by RT-PCR and sequencing (data not shown) using primers described previously [31,32]. It is likely the absence of associated bacteria explains why *P. tricornutum* has poor ability to use glucose or glycine.

### GL metabolism in *P. tricornutum*

Our results showed the growth rate under heterotrophic conditions was much lower compared to mixotrophic conditions (Figure 1C,I), indicating light was important for GL metabolism in *P. tricornutum*.

*In vivo*, GL can be phosphorylated and converted into dihydroxyacetone phosphate (DHAP) and glyceraldehyde 3-phosphate (GAP) through the following steps:

1. ATP + glycerol → ADP + sn-glycerol 3-phosphate
2. sn-glycerol-3-phosphate + NAD^+^ → DHAP + NADH + H^+^
3. DHAP → GAP
4. GAP → 3-P-glycerate (3PG)

and, finally, DHAP and 3PG can be metabolized through the glucolysis pathway. The complete genome sequence revealed *P. tricornutum* (CCMP 2561) contains all the enzymes required for the reactions mentioned above, that is, (1) glycerol kinase [gi: 7203857], (2) glycerol-3-phosphate dehydrogenase [gi: 7205122], (3) triosephosphate isomerase [gi: 7202457], and (4) glyceraldehyde-3-phosphate dehydrogenase [gi: 7203381] and phosphoglycerate kinase [gi: 7195255] [19], indicating *P. tricornutum* (CCMP 2561) can use GL as a carbon source.

The average FL value of protein amino acids was approximately 0.150 for the 30% (U-^13^C) labeling experiment (Table 1), indicating nearly half of the carbon came from CO_2_ fixation and inocula biomass. This suggested GL was assimilated simultaneously with CO_2_ fixation under mixotrophic conditions, which was likely why *P. tricornutum* under mixotrophic conditions had a higher growth rate compared to heterotrophic conditions. Nevertheless, since at least half of the carbon came from GL, the growth rate under heterotrophic conditions should be approximately half compared to mixotrophic conditions if the GL assimilated was independent of CO_2_ fixation and light. This was not the case, as suggested by Figure 1C,I, indicating light influences the assimilation of GL. Following the steps described above, metabolism of GL needs large amounts of ATP, which can be generated through photophosphorylation. Besides, DHAP and GAP generated from GL metabolism were intermediate metabolites of the Calvin cycle. It was likely that consumption of these metabolites through the Calvin cycle promotes GL metabolism in *P. tricornutum*. We focused on Ser, which had an unexpectedly low level of ^13^C enrichment at position 1 and was reported to be synthesized via the GOC cycle followed by photorespiratory reactions [26], to investigate other likely pathways for GL metabolism in *P. tricornutum*.

**Figure 4:**
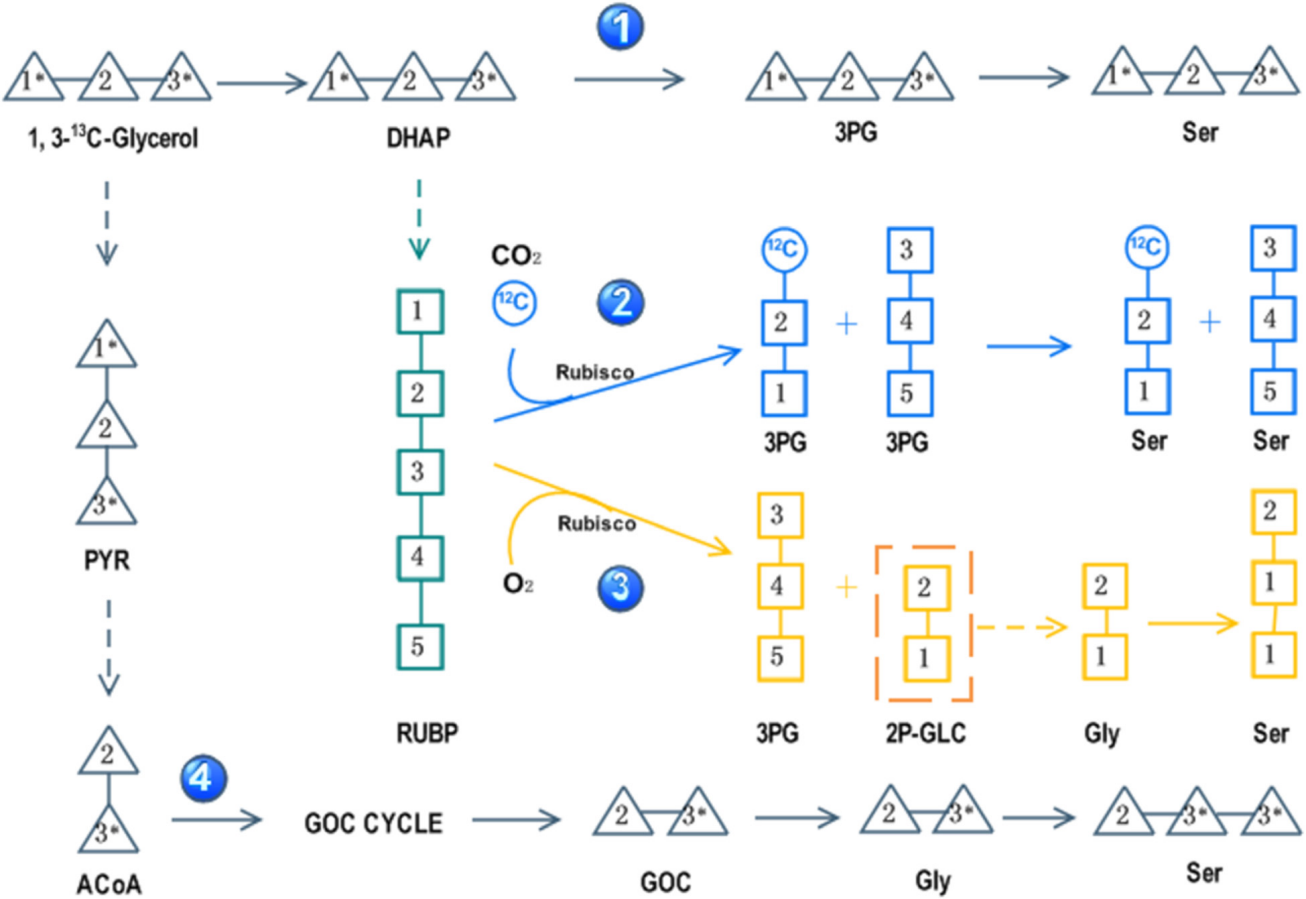
Scheme of four possible pathways for Ser arising from GL. (1) Ser was synthesized directly from GL-derived 3PG. The carbon backbone in GL was not disrupted and Ser had the same MDV as GL. (2) Ser was synthesized directly from Calvin cycle-derived 3PG, half of which would have an unlabeled carbon atom at position 1 deriving from atmospheric CO_2_. The ^13^C enrichment of Ser1 should be diluted regardless of the labeling pattern of GL. (3) Ser was synthesized from GOC through photorespiration. The carbon backbone in GL was recombined during the Calvin cycle and Ser1 was derived from RuBP2. (4) Ser was synthesized from GOC via the GOC cycle, as reported by Zheng *et al*. In this case, Ser1 originated solely from GL2 and the ^13^C enrichment should be zero when [1, 3-^13^C]GL was used as substrate.

As was reported, Ser can be synthesized directly from 3PG through a phosphorylated route [33] or from glycine by the glycolate pathway [34]. In *P. tricornutum*, Ser could be synthesized from GL by four routes (Figure 4): (1) Ser was synthesized directly from GL-derived 3PG through a phosphorylated route, in which case the carbon backbone in GL was not disrupted and Ser should have the same MDV as GL. (2) Ser was synthesized directly from the Calvin cycle-derived 3PG, half of which would have an unlabeled carbon atom derived from atmospheric CO_2_ in position 1. In this case, the ^13^C enrichment of Ser1 should be diluted by half regardless of the labeling pattern of GL. (3) Ser was synthesized from GOC through photorespiration (oxygenation of ribulose-1,5-bisphosphate, RuBP). (4) Ser was synthesized from GOC via the GOC cycle, as reported by Zheng *et al*. [26]; in this case, Ser1 origin solely from GL2, whose ^13^C enrichment should be zero using [1, 3-^13^C]GL as substrate. Our results showed the ^13^C enrichment of Ser1 was significantly lower (ca. half under normal condition and much lower under nitrogen-limited condition) compared to Ser2 and Ser3 when [1, 3-^13^C]GL was used as substrate and so was the ^13^C enrichment of Gly1 relative to Gly2 (Table 3). Nevertheless, both Ser and Gly had similar ^13^C enrichment in all positions when [U-^13^C]GL was used as substrate. Thus, it is unlikely Ser was synthesized solely from route 1, route 2 or route 4. It is more likely that Ser was largely synthesized, as in higher plants [34], from Gly via the photorespiratory pathway (route 3). In other words, the Calvin cycle and photorespiration took part in GL metabolism in *P. tricornutum*.

### The likely response of photorespiration to nitrogen limitation

It was reported that photorespiration was important for nitrogen assimilation [35]. To investigate the probable relationship between photorespiration and nitrogen assimilation in *P. tricornutum*, we labeled *P. tricornutum* in the absence of nitrogen using [U-^13^C]GL or [1,3-^13^C]GL as substrate. As Table 1 shows, the ^13^C enrichment of Ser and Gly were increased significantly when those of other amino acids decreased in the absence of nitrogen, regardless of the type of substrate. Synthesis of protein was reduced under nitrogen-limited conditions [36], meaning the *de novo* synthesis of amino acids would be reduced also. Thus, it was anticipated the ^13^C enrichment of most amino acids would be reduced. The high FL values of Ser and Gly under nitrogen-limited conditions suggested most Ser and Gly were synthesized *de novo* using carbon backbones obtained through GL metabolism. As mentioned above, Ser and Gly were synthesized through photorespiration (Figure 4, route 3); thus, the increase of Ser and Gly synthesis indicated the increase of photorespiration under nitrogen-limited conditions, consistent with the suggestion that photorespiration was important for nitrogen assimilation [35]. In addition, the response of diatom to nitrogen limitation was different from that of higher plants and green algae. Instead of photosynthesis, the breakdown of intracellular stores responded for carbon backbone demand for reassimilation of nitrogen [36]. We proposed that photorespiration might be important for redistribution of nitrogen under nitrogen-limited condition.

### Influence of sodium acetate on growth of *P. tricornutum* with or without nitrogen

It was reported photorespiration bypass can improve photosynthesis rates and biomass production in *Arabidopsis thaliana* [37,38]. One bypass was suggested by converting one molecule of GOC with one molecule of acetyl coenzyme A (ACoA) into malate by malate synthase [37-39]. Malate can be metabolized through other pathway (TCA cycle, GOC cycle, and so on) and thus reduce carbon loss through the photorespiratory pathway. To investigate likely photorespiration bypass and its influence on the growth of *P. tricornutum*, we compared the growth of *P. tricornutum* with or without sodium acetate, which can be converted into ACoA by ACoA synthase. The results indicated sodium acetate promoted the growth of *P. tricornutum* in the presence of nitrogen, but growth was decreased in the absence of nitrogen. We proposed that when sodium acetate was added, ACoA was synthesized by ACoA synthase. Excess ACoA promoted the condensation reaction between ACoA and GOC and resulted in the photorespiration bypass in *P. tricornutum*, which improved biomass production in the present of nitrogen. When nitrogen was limited, *P. tricornutum* had a slower growth rate in the presence of sodium acetate, suggesting photorespiration was important for the nitrogen-limited response in *P. tricornutum*, consistent with reports that photorespiration was important for nitrogen assimilation [35]. Together with the report that genes encoding GOC cycle-related enzymes were down-regulated significantly under nitrogen deprivation [40], we proposed down-regulation of the GOC cycle allows more GOC catalysis through photorespiration. The enhancement of photorespiration might facilitate the redistribution of nitrogen and thus helped *P. tricornutum* to survive under nitrogen-limited conditions. These results also provided evidences for GL metabolism through route 3 but not route 4 (Figure 4). In route 3, GOC was generated from oxygenation of RuBP. Excess ACoA would transfer GOC into malate, thus reduced carbon loss through following photorespiratory reactions, which would benefit growth under nitrogen-replete conditions but be unfavorable under nitrogen-limited conditions. While in route 4, glycerol was converted into ACoA and then GOC through the GOC cycle. GOC was metabolized through photorespiratory reactions. Excess ACoA would enhance route 4 (photorespiratory reactions), which should be favorable for growth under nitrogen-limited condition. This was not the case in our results; thus, we suggested that glycerol was largely metabolized through route 3 (Figure 4) in the strain used in this study.

### Metabolic analysis provided further evidences for the importance of photorespiration in nitrogen-limited response

To obtain further insight into the importance of photorespiration in nitrogen-limited response, the composition of the intracellular metabolite pool under different culture conditions (N^−^Ac^+^, N^−^Ac^−^, N^+^Ac^+^, and N^+^Ac^−^) was measured by NMR and the ratios of abundances of key cellular metabolites were calculated (Table 4). Gly increased under N^−^ conditions (N^−^Ac^+^/N^+^Ac^+^ and N^−^Ac^−^/N^+^Ac^−^) and was reduced when acetate was added (N^−^Ac^+^/N^−^Ac^−^ and N^+^Ac^+^/N^+^Ac^−^). In addition, ethylene glycol and formate, which can be produced from intermediate metabolites of photorespiration (glycolate → glycolaldehyde → ethylene glycol and glyoxylate → oxalate → formate), had the same expression pattern as Gly (Table 4). These results supported our hypotheses that photorespiration was up-regulated under N^−^ condition, whereas addition of acetate would result in photorespiration bypass and suppress the following steps of photorespiration. Besides, Glu and Gln, which participated in photorespiration as nitrogen carriers, were reduced under N^−^ conditions (N^−^Ac^+^/N^+^Ac^+^ and N^−^Ac^−^/N^+^Ac^−^) and increased when acetate was added (N^−^Ac^+^/N^−^Ac^−^ and N^+^Ac^+^/N^+^Ac^−^). Accordingly, AKG (the precursor of Glu) increased under N^−^ conditions (N^−^Ac^+^/N^+^Ac^+^ and N^−^Ac^−^/N^+^Ac^−^) and was reduced when acetate was added (N^−^Ac^+^/N^−^Ac^−^ and N^+^Ac^+^/N^+^Ac^−^). In theory, Glu and Gln might have the same expression pattern as Gly (increased under N^−^ conditions). Yet Glu and Gln also played as nitrogen carriers for biosynthesis. Since cell growth and most amino acids were reduced under nitrogen-limited condition (Figure 1D and Table 4), it was probable that an approximately 0.25-fold amount of Glu was sufficient to support photorespiration.

### Likely GL metabolism pathway in *P. tricornutum*

**Figure 5:**
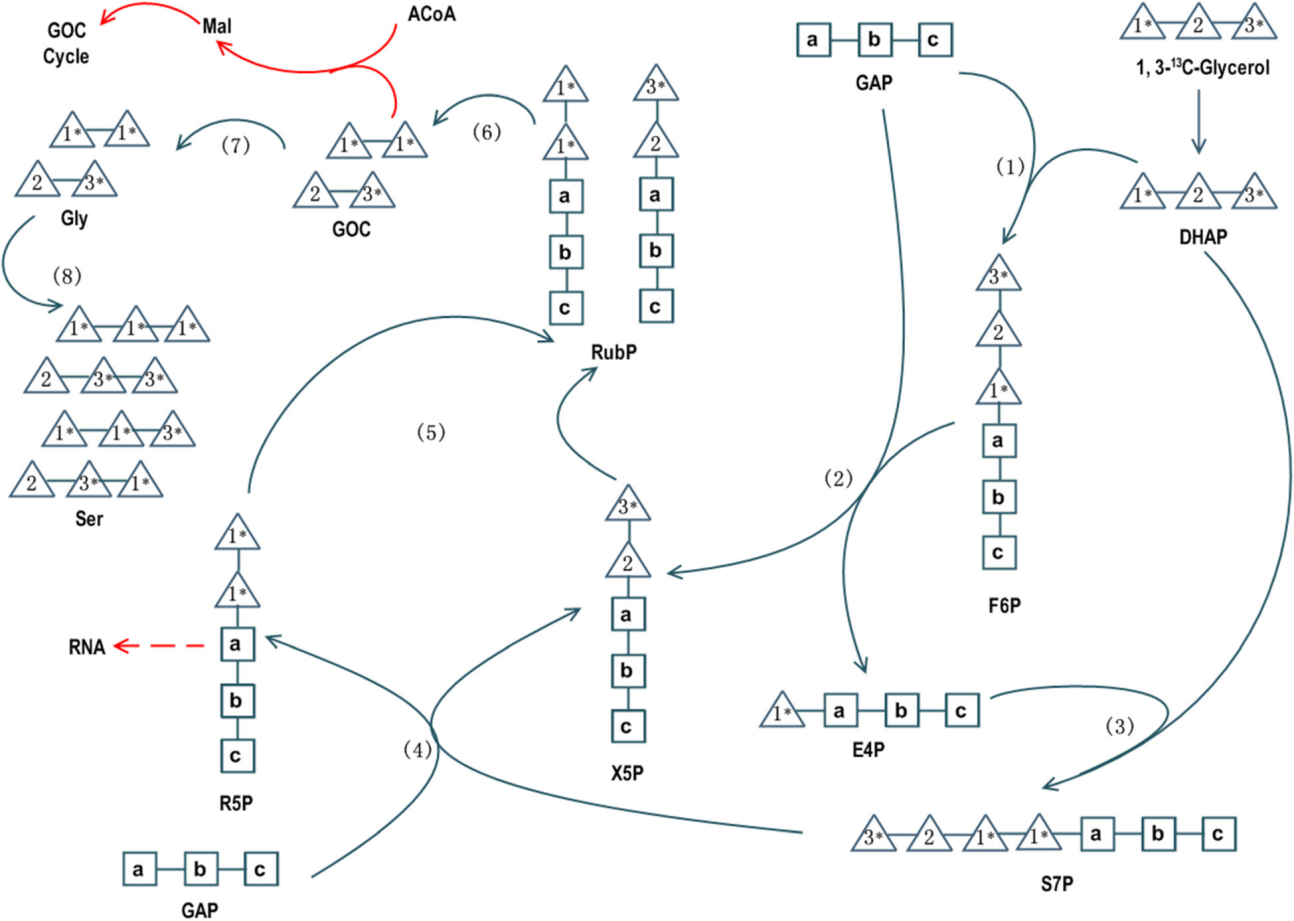
Scheme of possible pathways for GL metabolism in *P. tricornutum*. (1) One molecule of F6P was derived through a condensation reaction between one molecule of GAP and one molecule of DHAP. Carbon backbone from GL3_1 was distributed at F6P1_3. (2) F6P1_2 was transferred to a second molecule of GAP, generating one molecule of X5P with GL3_2-generated carbon backbone distributed at X5P1_2 and one molecule of E4P with GL1-derived carbon atom distributed at E4P1. (3) E4P condensate with a second molecule of DHAP, generating one molecule of S7P with GL1-derived carbon atom distributed at S7P4 and GL3_1-derived carbon backbone distributed at S7P1_3. (4) S7P1_2 were transferred to a third molecule of GAP, generating one molecule of X5P with GL3_2-generated carbon backbone distributed at X5P1_2 and one molecule of R5P with GL1-derived carbon atoms distributed at R5P1 and R5P2. (5) RuBP generated from R5P and X5P. (6) Generation of GOC (GOC) from oxygenation of RuBP. (7) Generation of Gly from GOC. A third of Gly had carbon atoms generated from GL1 at both positions, and others had GL2 at Gly1 and GL3 at Gly2. (8) Generation of Ser from Gly. Ser had GL1 or GL2-derived carbon atom at Ser1 and GL1 or GL3 at Ser2 and Ser3.

Notably, the FL values of Ser and Gly in the absence of nitrogen based on [U-^13^C]GL were approximately 0.3, close to the level of substrate. During photorespiration, 2P-glycolate is generated from oxygenation of RuBP, whose ^13^C enrichment was inevitably diluted by CO_2_ during the Calvin cycle. Since Ser and Gly were synthesized from GOC, which was generated from 2P-glycolate, why were they not diluted by CO_2_? Obviously, only when the carbon atoms in position 1 and position 2 of pentose-5-phosphate (P5P1 and P5P2) were derived solely from GL could GOC retain a high level of ^13^C enrichment. This cannot be the case when GL entered the Calvin cycle as GAP, as GAP can also be derived from carboxylation of RuBP whose ^13^C enrichment would be diluted. Thus, GL likely entered the Calvin cycle in another form, for example, DHAP. Supposing all DHAP was generated from GL (Figure 5), RuBP regeneration started with the condensation reaction of one molecule of GAP and one molecule of DHAP, generating one molecule of fructoce-6-P (F6P) with GL3_1-generated carbon atoms at F6P1_3. Next, F6P1_2 was transferred to a second molecule of GAP, generating one molecule of xylulose-5-P (X5P) with the GL 3_2-generated carbon backbone at X5P1_2 and one molecule of erythrose-4-P (E4P) with a GL1-derived carbon atom distributed at E4P1. Next, E4P was condensed with a second molecule of DHAP, generating one molecule of sedoheptulose-7-P (S7P) with a GL1-derived carbon atom distributed at S7P4 and the GL3_1-derived carbon backbone distributed at S7P1_3. Sequentially, S7P1_2 was transferred to a third molecule of GAP, generating one molecule of X5P with a GL3_2-generated carbon backbone distributed at X5P1_2 and one molecule of ribose-5-P (R5P) with GL1-derived carbon atoms distributed at R5P1 and R5P2. Both R5P and X5P can be converted into ribulose-5-P (Ru5P), which can finally be converted into RuBP. By oxygenation by Rubisco and metabolism through photorespiration, these RuBPs can generate three molecules of Gly: one had carbon atoms generated from GL1 at both positions and the other two had GL2 at Gly1 and GL3 at Gly2. Generated from these Gly, Ser would have a GL1 or GL2-derived carbon atom at Ser1 and GL1 or GL3 at Ser2 and Ser3. In this pathway, Ser and Gly would have a low level of ^13^C enrichment at position 1 and similar high level of ^13^C enrichment at other positions. As GL1-derived Ser1 and Gly1 are basically derived from R5P, the ^13^C enrichment of Gly1 and Ser1 would depend on the ratio of R5P to X5P used in the generation of RuBP. When more R5P is used, a higher ^13^C enrichment at Gly1 and Ser1 would occur. This might account for the different ^13^C enrichment in Gly1 and Ser1 under different nitrogen conditions, providing further evidence for GL metabolism through route 3. Ser1 and Gly1 were significantly lower under nitrogen-limited conditions compared to when ample nitrogen was present, indicating the proportion of RuBP generated from R5P decreased. It was probable that R5P was used for synthesis of other compounds, for example, mRNA. *P. tricornutum* might reserve mRNA synthesis under nitrogen-limited conditions to prepare for recovery under optimized conditions, consistent with our results for transcriptome and proteome of *P. tricornutum* under nitrogen-limited conditions (unpublished data).

## Conclusion

Our results indicated the strain used in this study (IOCAS-001) was different from that in the study of Zheng *et al.* [26]. Strains that lack a glucose-utilizing ability have a different mechanism for organic carbon metabolism from strains that can use glucose. Photorespiration is involved in GL metabolism and is important for the nitrogen-limited response in *P. tricornutum*.

## Materials and methods

### Strains and culture conditions

Axenic cultures of *P. tricornutum* were screened from the East China Sea and maintained in our laboratory for decades and identified as *P. tricornutum* (IOCAS-001) according to morphological observation and the 18s ribosomal RNA sequence. The cultures were grown in f/2 medium [41] made with steam-sterilized NaHCO_3_-free artificial seawater [42] supplemented with f/2 inorganic nutrients, trace elements and vitamins (sterilized by filtration). One volume of cells in mid-exponential growth phase was harvested by centrifugation for 10 min at 5000×*g*, washed with sterilized NaHCO_3_-free artificial seawater, and inoculated into nine volumes of fresh medium. Then, 100 mg · L^−1^ each of ampicillin, kanamycin, and streptomycin were added to avoid bacterial contamination. NaHCO_3_ (0.174 g·L^−1^) or a source of organic carbon (see below) was added. For nitrogen-free experiments, sodium nitrate was omitted from f/2 inorganic nutrients. For experiments testing the influence of sodium acetate, it was added to a final concentration of 1 g·L^−1^. To further test the influence of sodium acetate concentration, it was added to final concentrations of 0, 0.1, 0.5, 1, and 5 g·L^−1^, respectively. Each treatment was conducted in triplicate. Cultures were grown at 20°C under cool white fluorescent lights at approximately 100 μmol m^−2^·s^−1^ with a 12 h dark/12 h light cycle for mixotrophic conditions and completely dark for heterotrophic conditions. Cell growth was monitored by measurement of absorbance at 730 nm (*A*_730nm_) using a UV/visible spectrophotometer (Uv-1800; Shimadzu Scientific Instruments, Japan). Cell number was counted using a haemacytometer, and their relationship with the value of *A*_*730nm*_ was determined.

### Selection of carbon source and optimization of GL concentration

Cells were cultured with 0.02 M GL, glucose, or glycine as carbon source. To determine the optimal concentration of GL, cells were cultured with 0.005, 0.02, and 0.1 M GL as carbon source. *A*_730nm_ was measured and compared to growth with no additional carbon source and with NaHCO_3_ at a final concentration of 0.174 g·L^−1^ as carbon source.

### Determination of GL and acetate concentrations

The culture was centrifuged at 8,000×*g* for 10 min at room temperature. The supernatant was used to detect extracellular metabolites by LC as described previously [43]. Basically, 10 μL sample were loaded onto a Bio-Rad HPX-87H column (Bio-Rad; Hercules, CA, USA) connected to an HPLC system (Agilent 1200) (GMI; Ramsey, MN, USA), eluted with 5 mM H_2_SO_4_ at a flow rate of 0.5 mL·min^−1^, and then detected with a differential refraction detector.

### Total lipid analysis

Cells were harvested and quickly froze in liquid nitrogen and then freeze-dried. Total lipids were extracted as described by Bligh and Dyer [44] with minor modifications. Briefly, approximately 20 mg dried cells were mixed with 1 mL of chloroform/methanol (1:1). The mixture was stirred vigorously for 5 min, after which 0.3 mL of 0.2 M H_3_PO_4_ (containing 1 M KCl) was added and mixed. After centrifugation at 5000 × g for 5 min, the solvent phase was recovered. This process was repeated three times. The solvent phases were combined, washed three times with distilled water, and then evaporated at room temperature under a ventilated fume hood. The total lipid content was obtained by weighing and expressed as percent cell dry weight (% CDW).

### ^13^C labeling experiment

GL (0.02 M) was used as the ^13^C-labeling substrate. Mid-exponential growth phase cells (with 0.174 g·L^−1^ NaHCO_3_ or 0.02 M GL as carbon source) were harvested by centrifugation for 10 min at 5000×*g*, washed with sterilized NaHCO_3_-free artificial seawater, and inoculated into nine volumes of fresh medium containing a 20%:80% (or 30%:70%) *w*/*w* mixture of [U-^13^C]GL (99%; Cambridge isotope laboratories)/unlabeled GL or 50%:50% w/w mixture of [1,3-^13^C]GL (99%; Cambridge isotope laboratories)/unlabeled GL. For labeling under N-limited conditions, NaNO_3_ was omitted from the medium. The labeling experiment lasted for 10 days under normal conditions and a week under N-limited conditions when the cells grew to mid-exponential growth phase. Each treatment was conducted in triplicate.

### Sample preparation and gas chromatography-mass spectrometry analysis

Samples for GC-MS analysis were prepared as described previously [27]. After labeling, cells were harvested by centrifugation for 10 min at 5000×*g*, washed with distilled water, and placed into 1.5 mL Eppendorf tubes and centrifuged for 5 min at 10,000×*g*. The supernatants were discarded, and cell pellets were hydrolyzed in 6 M HCl at approximately 110°C for 12 h then dried overnight at approximately 80°C. Subsequently, each dried sample was dissolved in 100 μL of water-free pyridine then derivatized by the addition of 50 μL of *N*-tert-butyldimethylsilyl-*N*-methyltrifluoroacetamide (MTBSTFA) and heated at 85°C for 60 min. The sample was centrifuged for 5 min at 10,000×*g*, and the supernatant was filtered through 0.22-μm pore-size filters and then loaded onto a GC-MS apparatus for analysis as previously described [45]. GC-MS was performed with an Agilent 6890-5973 GC-MS (Conquer Scientific; San Diego, CA, USA) system equipped with an Agilent HP-5MS column (30 m × 0.25 mm × 0.25 μm) with helium as the carrier gas. The oven temperature was programmed at 100°C for 2 min then increased to 260°C at a rate of 5°C min^−1^ and kept at 260°C for 10 min. The injection volume was 2 mL, and the MS device was operated in scan mode.

### GC-MS data processing

The GC-MS data were processed as previously described [27]. The mass distribution vector (MDV) for each fragment of 13 amino acid residues (Ala, Gly, Val, Asx, Glx, Pro, His, Phe, Tyr, Ser, Thr, Lys, and Met) and GL were assigned according to Eq. (1), where *m*_0_ is the fractional abundance of molecules with monoisotopic mass and *m*_*i*>0_ is the abundance of fragments with greater mass [46] and corrected for the natural abundance of stable isotopes of O, N, Si, S, H, and C [27].

1 $$ MD{V}_a=\left[\begin{array}{l}\left({m}_0\right)\hfill \\ {}\left({m}_1\right)\hfill \\ {}\kern1em \vdots \hfill \\ {}\left({m}_n\right)\hfill \end{array}\right]\mathrm{with}{\displaystyle {\sum}_{i=0}^n{m}_i=1} $$

The fractional labeling (FL) of each fragment was calculated according to Eq. (2), where *mi* has the same meaning as Eq. (1) [45].

2 $$ \mathrm{F}\mathrm{L}=\frac{{\displaystyle {\sum}_{i=0}^ni\cdot {m}_i}}{n\cdot {\displaystyle {\sum}_{i=0}^n{m}_i}} $$

### Metabolite analysis

Cells cultured under N^−^Ac^+^, N^−^Ac^−^, N^+^Ac^+^, and N^+^Ac^−^ conditions were harvested and quickly frozen in liquid nitrogen, then freeze-dried. Metabolites were extracted and analysis by NMR as described previously [47]. Student *t* test was used to detect significance of differences between samples.

## Additional file

Additional file 1: Figure S1. Growth of *P. tricornutum* on NH_4_Cl and NaNO_3_ as nitrogen sources.

## Abbreviations

PUFA: polyunsaturated fatty acids
GC-MS: gas chromatography mass spectrometry
NMR: nuclear magnetic resonance
GL: glycerol
CDW: cell dry weight
FL: fractional labeling
PEP: phosphoenolpyruvate
E4P: erythrose-4-P
MDV: mass distribution vector
DHAP: dihydroxyacetone phosphate
GAP: glyceraldehyde 3-phosphate
3PG: 3-P-glycerate
RuBP: ribulose-1,5-bisphosphate
ACoA: acetyl coenzyme A
P5P: pentose-5-phosphate
F6P: fructoce-6-P
X5P: xylulose-5-P
E4P: erythrose-4-P
S7P: sedoheptulose-7-P
R5P: ribose-5-P
Ru5P: ribulose-5-P
MTBSTFA: *N*-tert-butyldimethylsilyl-*N*-methyltrifluoroacetamide
